# Biological characteristics of Rh123^high^ stem-like cells in a side population of 786-O renal carcinoma cells

**DOI:** 10.3892/ol.2013.1270

**Published:** 2013-03-26

**Authors:** JIANZHONG LU, YONG CUI, JIAYUN ZHU, JINPENG HE, GUANGMING ZHOU, ZHONGJIN YUE

**Affiliations:** 1 Institute of Urology, The Second Hospital of Lanzhou University, Key Laboratory of Urological Diseases in Gansu Province, Gansu Nephro-Urological Clinical Center, Lanzhou, Gansu 730030;; 2 Institute of Modern Physics, Radiobiological Effects Group, Chinese Academy of Sciences, Lanzhou, Gansu 730000, P.R. China

**Keywords:** rhodamine 123, cancer stem cell, renal cell carcinoma, flow cytometery, tumorigenesis

## Abstract

The aim of the present study was to investigate the differences in biological characteristics between the rhodamine 123 (Rh123)^high^ and Rh123^low^ subpopulations of the renal cancer cell line 786-O and to identify evidence for the existence of cancer stem cells in renal cell carcinoma (RCC) cells. *In vitro* cultured RCC 786-O cells were stained with Rh123, analyzed and sorted with flow cytometry. The differences in proliferative activity, long-term differentiation and radiation sensitivity between the two subpopulations were measured and the oncogenicity of each subpopulation was evaluated according to their neoplastic growth ability in soft agar and tumor-forming ability in NOD/SCID immunodeficient mice. There were two subpopulations in the cultured 786-O cells, Rh123^high^ and Rh123^low^ cells. Rh123^low^ cells were the majority among 786-O renal carcinoma cells and barely formed solid tumors in NOD/SCID mice and colonies in soft agar. By contrast, the Rh123^high^ cells were the minority, exhibited high proliferative activity, differentiation ability and resistance to radiation and showed high tumorigenesis potential and colony forming efficiency. The Rh123^high^ cells had stem-like characteristics in cultured RCC 786-O cells *in vitro*.

## Introduction

Cancer stem cells (CSC), which have the ability to self-renew and differentiate, may contribute to tumor proliferation and recurrence following chemotherapy and radiotherapy. Currently, CSCs have become a key focus of tumor studies since they are expected to reveal biomarkers which may be *de novo* targets for tumor therapy. CSCs have already been identified in numerous tumor types, including breast (1), brain (2), colon (3,4) and prostate cancers (5).

The ability of stem cells to efflux chemotherapeutic drugs and certain dyes, such as Hoechst 33342 and rhodamine 123 (Rh123) (6), may be used to isolate cells with progenitor characteristics (7,8). Cells stained with such fluorescent dyes may present distinguishable subpopulations in flow cytometry profiles, allowing sorting for further biological studies. This technique provides an alternative approach to isolating progenitor cells through the use of specific surface markers and a feasible method for identifying putative tumor-initiating cells.

Rh123, a low toxicity fluorescent dye, is a mitochondrial dye that stains mitochondria with increasing intensity as cells become activated. It is able to detect reduced mitochondrial activation states in long-term quiescent cells. Decreased intracellular accumulations of Rh123 result from the efflux of the dye (9).

Renal cell carcinoma (RCC) is a kidney cancer that originates from the proximal convoluted tubule. It is the most common type of kidney cancer in adults, responsible for ∼80% of cases (10). It is also known to be the most lethal of all the genitourinary tumors. RCC is resistant to radiation therapy and chemotherapy (11). Thus, it is necessary to identify a CSC subpopulation in RCC, since screening and identifying RCC stem cells is likely to be of significance for prognosis and treatment. However, it is not yet clear whether there are CSCs in renal carcinoma. The aim of the present study was to isolate cancer stem-like cells from the renal carcinoma cell line 786-O using Rh123 staining and flow cytometry, and compare the various biological characteristics between subpopulations.

## Materials and methods

### Cell line and culture

The human renal cancer cell line 786-O was purchased from the Shanghai Cell Bank of Type Culture Collection (Chinese Academy of Sciences, Shanghai, China) and maintained in RPMI-1640 medium (Gibco, Grand Island, NY, USA) containing 10% heat-inactivated fetal bovine serum (FBS; Hyclone, Waltham, MA, USA), 100 U/ml penicillin G and 100 *μ*g/ml streptomycin in a humidified 5% CO_2_ incubator at 37°C.

### Sorting side population (SP) cells

Once the 786-O cells had reached the logarithmic growth phase, they were harvested for flow cytometry. Briefly, cells were digested with 0.25% trypsin (Sigma-Aldrich, St. Louis, MO, USA), washed twice with calcium/magnesium-free phosphate-buffered saline (PBS), resuspended in ice-cold RPMI-1640 supplemented with 5% FBS at a concentration of 1×10^6^ cells/ml and incubated at 37°C in a 5% CO_2_ incubator for 10 min. The fluorescent dye, Rh123 (Sigma-Aldrich), was then added at a final concentration of 10 *μ*g/ml and incubated for 20 min in the dark with periodic mixing. The cells were washed twice with PBS, then kept at 4°C in the dark before being subjected to the flow cytometery assay and sorting using a FACSCalibur flow cytometer (BD Biosciences, Mountain View, CA, USA). The Rh123^high^ and Rh123^low^ cells were collected to evaluate the sorting purity and perform further experiments.

### Cell growth

Freshly sorted Rh123^high^ and Rh123^low^ cells were incubated at 2×10^3^ cells per well in 12-well plates and cultured in complete RPMI-1640 medium to observe the growth rate. During the 10 days, the cells were photographed and the numbers of cells were counted using a hemacytometer.

### Colony formation

Freshly sorted cells (1×10^3^; Rh123^high^ and Rh123^low^ cells) were suspended in 2 ml of 0.35% melted agar in RPMI-1640 medium with 10% FBS and plated in 60-mm dishes containing a solidified bottom layer of 1.25% agar in the same medium. After 3 weeks, the number of colonies containing >50 cells was counted. The colony-forming efficiency (CFE) was calculated as the ratio of the colony number to the original number of cells seeded. The experiments were independently performed three times.

### Long-term differentiation of Rh123^high^ and Rh123^low^ cells

The Rh123^high^ and Rh123^low^ cells were subcultured for 18 days after cell sorting. The cells were then stained separately with Rh123 and analyzed with a flow cytometer to quantitate the proportion of the Rh123^high^ subpopulation in each sorted group.

### Radiation sensitivity assay

Freshly sorted Rh123^high^ and Rh123^low^ cells (2×10^5^) were seeded in five culture flasks and the cells were irradiated with 0, 0.5, 1, 2 and 4 Gy of X-rays, The next day after radiation treatment, the cells were then harvested and seeded at various low densities into 35-mm dishes. All the cells were cultured under normal culture conditions (humidified 5% CO_2_ incubator at 37°C). After 10 days, when the majority of cell clones in the sham control group reached >50 cells, the dishes were stained with crystal violet to determine the plating efficiency. The experiments were independently performed three times.

### Xenograft tumor

The nonobese diabetic/severe combined immunodeficient (NOD/SCID) mice were purchased from Shanghai Slac Laboratory Animal Co. Ltd. (Shanghai, China) and maintained in micro isolator cages. All experiments were approved by the animal care committee of the Second Hospital of Lanzhou University. Freshly sorted Rh123^high^ and Rh123^low^ cells (1×10^7^, 1×10^6^ or 1×10^5^ cells/ml) were suspended in 200 *μ*l PBS and then inoculated into the axillary fossa of six-week-old male NOD/SCID mice immediately after cell sorting. The mice were monitored twice a week for palpable tumor formation and euthanized when the tumors grew to appropriate sizes. Tumors were measured using a vernier caliper, weighed and photographed.

### Cell surface marker screening

Freshly sorted Rh123^high^ and Rh123^low^ cells were incubated in RPMI-1640 for 2 days to reach the desired cell number. Then, the cultured cells were harvested and diluted to 1×10^6^ cells/ml with PBS containing 5% FBS, and stained with Rh123 for 20 mins. The Rh123 positive staining cells were divided into multiple vials and each was probed with CD3-FITC, CD4-PE, CD8-PerCP, CD24-PE and CD44-FITC antibody for 30 min in the dark, then analyzed by FACS to quantitate the proportion of positive cells.

### Statistical analysis

The SPSS 12.0 statistical software (SPSS Inc., Chicago, IL, USA) was used for the data processing and analyzing the significance of differences between the Rh123^high^ and Rh123^low^ cells using unpaired t-tests. P<0.05 was considered to indicate statistically significant differences. Data were expressed as the mean ± SD from at least three independent experiments.

## Results

### Rh123 staining and sorting of 786-O cells

**Figure 1:**
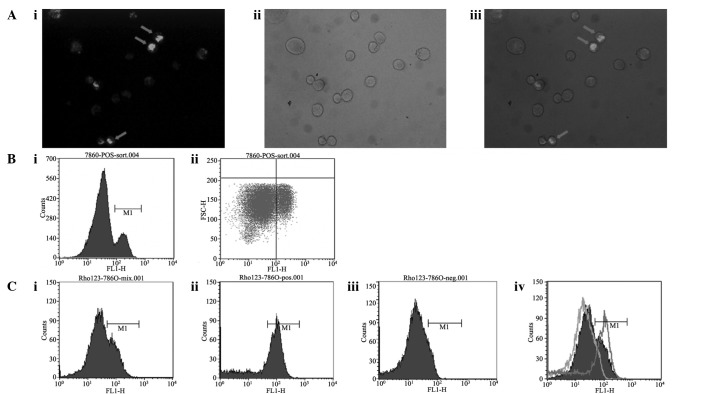
786-O cells were stained with Rh123 and sorted by flow cytometry. (A) After staining, 786-O cells were observed under a fluorescence microscope.The Rh123^high^ cells were markedly stained (arrows). (i) Rh123 staining; (ii) phase-contrast image; (iii) merged image of (i) and (ii). (B) Analysis with FACSCalibur flow cytometry with the FL1 channel. Cells were separated into two distinguishable sub-populations: (i) histogram profile; (ii) dot profile. Gate M1 was set up to sort Rh123^high^ cells. (C) 786-O cells were sorted following Rh123 staining and two distinguishable sub-populations (Rh123^high^ and Rh123^low^) were observed in the flow cytometry profile and collected separately. Immediately after sorting, the purity of each sorted population was verified by flow cytometry analysis. (i) Fluorescence distribution prior to sorting; (ii) sorted Rh123^high^ cells; (iii) sorted Rh123^low^ cells; (iv) overlay of sorted and unsorted cells. Rh123, rhodamine 123.

786-O cells stained with Rh123 were observed under a fluorescence microscope. It was observed that a small fraction of the cells were stained green, namely the Rh123^high^ cells, while the majority presented no or extremely weak fluorescence, namely the Rh123^low^ cells (Fig. 1A). In the flow cytometry profile, two distinguishable subpopulations were observed, Rh123^high^ and Rh123^low^ (Fig. 1B). The proportion of Rh123^high^ cells was 25.17±3.88%. Gate M1 was set up to sort the Rh123^high^ cells and the results are shown in Fig. 1C. The Rh123^low^ and Rh123^high^ cells were each collected for subsequent experiments. The purity of the Rh123^high^ cells was 67.40% and the purity of the Rh123^low^ cells was 91.12%.

### Cell growth and colony formation

**Figure 2:**
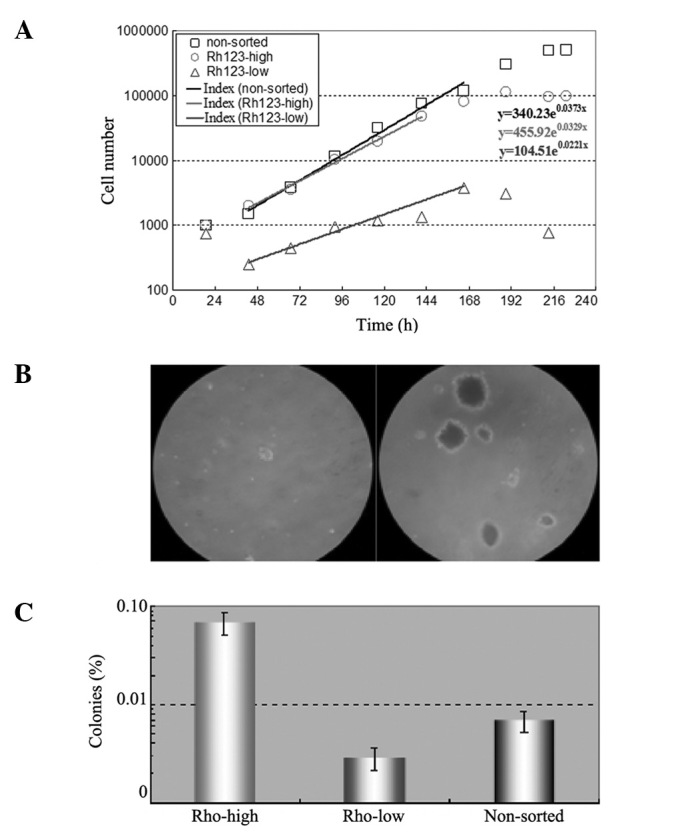
Growth curve and clone formation of Rh123^high^, Rh123^low^ and unsorted 786-O cells. (A) Growth curve of Rh123^high^, Rh123^low^ and unsorted 786-O cells. (B) Sorted colonies of Rh123^low^ cells (Left) and Rh123^high^ cells (Right) in soft agar. (C) Colony formation efficiency of Rh123^high^, Rh123^low^ and unsorted 786-O cells. Rh123, rhodamine 123.

The same number of sorted Rh123^low^ and Rh123^high^ cells and unsorted cells were seeded in 12-well plates and counted each day for up to 12 days. The results are shown in Fig. 2A. The Rh123^high^ cells exhibited a similar proliferation rate to the unsorted cells and their doubling times were 21 and 18 h, respectively. However, the Rh123^low^ cells grew slowly and with a doubling time of 36 h.

In the colony formation assay, the same number of the three types of cells were seeded in soft agar and cultured for >3 weeks prior to colony counting. The results are shown in Fig. 2B and C. The majority of Rh123^high^ cell colonies were mulberry-like in appearance and the colony-forming efficiency (CFE) was 0.687±0.177%, while the Rh123^low^ cells did not produce significant cell colonies and their CFE was only 0.029±0.007%.

### Long-term differentiation ability

**Figure 3:**
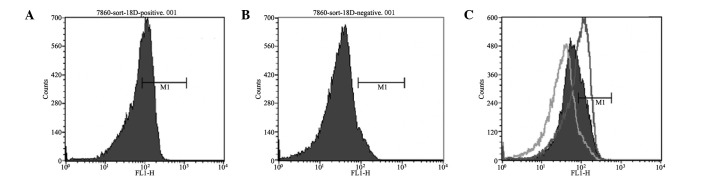
Cell differentiation potential and self-renewal ability. Sorted cells were subsequentially cultured for up to 18 days, then subjected to Rh123 staining and flow cytometry analysis. (A) Proportion of Rh123^high^ cells declined although a considerable number of the Rh123^high^ cells remained. (B) After continued subculture of Rh123^low^ cells, only Rh123^low^ cells were observed. (C) Combination of A, B and unsorted cells. Rh123, rhodamine 123.

The Rh123^high^ cells had a higher differentiation potential than the Rh123^low^ cells. As shown in Fig. 3, the proportion of Rh123^high^ cells in the sorted Rh123^high^ subpopulation decreased with subculture time following sorting, while there was no Rh123^high^ subpopulation among the subcultured Rh123^low^ cells.

### Tumor formation

**Figure 4:**
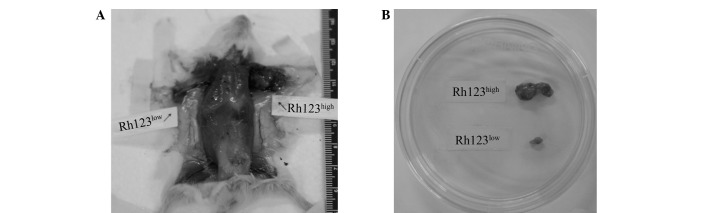
Tumor formation in NOD/SCID mice. Rh123, rhodamine 123; NOD/SCID, nonobese diabetic/severe combined immunodeficient.

Solid tumors in NOD/SCID immunodeficient mice implanted with 1×10^7^, 1×10^6^ or 1×10^5^ Rh123^high^ and Rh123^low^ cells were examined twice a week. Tumors were observed in 12 out of 12 mice injected with 1×10^7^, 1×10^6^ and 1×10^5^ Rh123^high^ cells at days 8, 20 and 90. However, almost no visible tumors were observed in the mice implanted with corresponding amounts of Rh123^low^ cells and only one out of the 12 mice injected with Rh123^low^ cells developed a tumor (Fig. 4).

### Radiosensitivity

**Figure 5:**
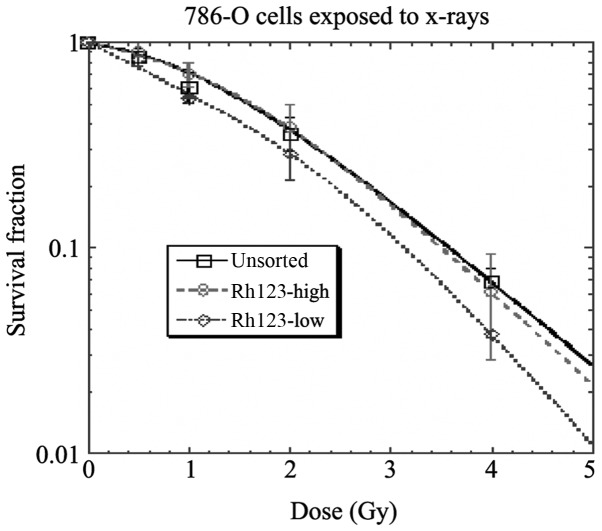
Sorted and unsorted cells were treated with 6 MV X-ray irradiation. Cells were collected, diluted and plated at low density into 35-mm dishes. After 10 days, the cells were fixed, stained and counted. Each experiment was independently repeated three times. Rh123, rhodamine 123.

When exposed to X-rays, the Rh123^high^ cells behaved similarly to unsorted cells and showed noticeably larger survival fractions compared with the Rh123^low^ cells (Fig. 5).

### Screening of surface markers

**Figure 6:**
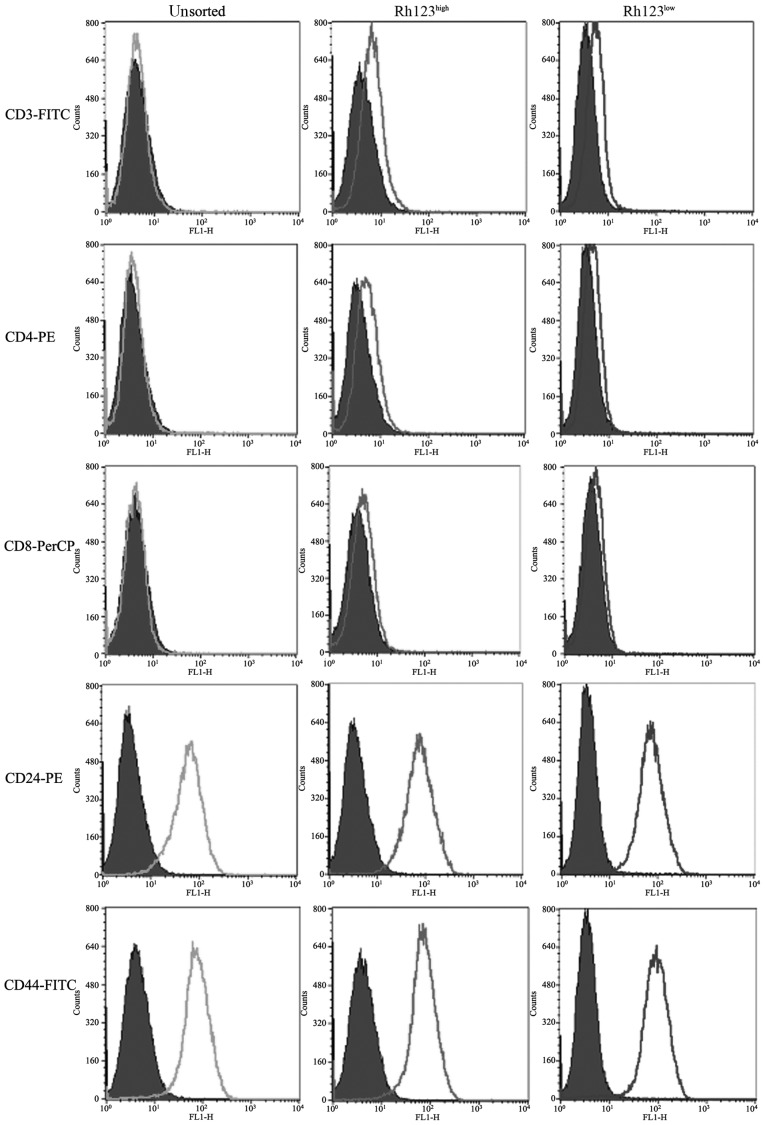
Screening for cell surface markers. Sorted and unsorted cells were stained with various fluorescence-labelled antibodies and analyzed with FACS. The light grey line in each plot was the unlabeled control. FACS, fluorescence-activated cell sorting; FITC, fluorescein isothiocyanate; Rh123, rhodamine 123.

As shown in Fig. 6, the two cell subpopulations and unsorted cells were positive for CD24 and CD44, but negative for CD3, CD4 and CD8. These cell surface markers were clearly not specific markers for RCC stem cells.

## Discussion

RCC is a difficult malignancy to treat due to its ability to spread asymptomatically and its inherent resistance to radiotherapy and chemotherapy (12). The identification of RCC stem cells is expected to provide a novel approach to solving this problem.

In the present study, cancer stem-like SPs were successfully derived from the human renal cancer cell line 786-O, using rhodamine 123 staining and sorting by flow cytometry. Notably, the Rh123^high^ SP cells were observed to have the basic characteristics of CSCs. It is generally considered that CSCs exist in Rh123^low^ subgroups (13,14), although the present results demonstrated the opposite of this. By using flow cytometry after Rh123 staining, human cervical carcinoma HeLa, hepatoma HepG2, melanoma OCM-1 and gastric cancer MGC-803 cells were analyzed and the results were consistent with the published literature. The proportion of Rh123^low^ cells in each cell line was <5% (data not shown). However, human renal cancer cells were different from these tumors. In *in vitro* cultured RCC 786-O cells, the Rh123^high^ SP cells were less common than the Rh123^low^ subgroup and the tumorigenic capacity of Rh123^low^ SP cells was less than that of the Rh123^high^ cells. These results suggest that the biological characteristics of RCC may differ from carcinomas originating from other tissues.

CSCs theoretically have unlimited proliferative ability, self-renewal capacity and multi-differentiation potential, which drives tumor formation and growth (13). In the present study, the Rh123^high^ subpopulation of RCC 786-O cells was demonstrated to be capable of driving tumor formation and growth. The growth characteristics (Fig. 2), tumorigenesis (Fig. 3), differentiation potential (Fig. 4) and radiotherapy resistance (Fig. 5) suggested that the Rh123^high^ subpopulation has RCC stem cell-like characteristics.

Bussolati *et al*(15) observed that the mesenchymal stem cell marker CD105-positive cells present in human renal carcinomas were the renal tumor-initiating cell population. However, CD105 expression was not observed on the surface of 786-O cells (data not shown). Pode-Shakked (16) observed that NCAM was a putative marker for the Wilms’ tumor stem/progenitor cell population, although we have not studied whether NCAM is expressed in RCC cells. In the present study, the cell surface markers CD3, CD4, CD8, CD24 and CD44 were excluded as stem cell markers for RCC (Fig. 6).

Further characterization of the RCC Rh123^high^ cells, by studying clinical RCC specimens and screening for surface markers, is likely to have an impact on their clinical application.
